# Selection of Patients and Anesthetic Types for Endovascular Treatment in Acute Ischemic Stroke: A Meta-Analysis of Randomized Controlled Trials

**DOI:** 10.1371/journal.pone.0151210

**Published:** 2016-03-08

**Authors:** Fubing Ouyang, Yicong Chen, Yuhui Zhao, Ge Dang, Jiahui Liang, Jinsheng Zeng

**Affiliations:** Department of Neurology and Stroke Center, the First Affiliated Hospital of Sun Yat–Sen University, Guangzhou, 510080, China; St Michael's Hospital, University of Toronto, CANADA

## Abstract

**Background:**

and Purpose Recent randomized controlled trials have demonstrated consistent effectiveness of endovascular treatment (EVT) for acute ischemic stroke, leading to update on stroke management guidelines. We conducted this meta-analysis to assess the efficacy and safety of EVT overall and in subgroups stratified by age, baseline stroke severity, brain imaging feature, and anesthetic type.

**Methods:**

Published randomized controlled trials comparing EVT and standard medical care alone were evaluated. The measured outcomes were 90-day functional independence (modified Rankin Scale ≤2), all-cause mortality, and symptomatic intracranial hemorrhage.

**Results:**

Nine trials enrolling 2476 patients were included (1338 EVT, 1138 standard medical care alone). For patients with large vessel occlusions confirmed by noninvasive vessel imaging, EVT yielded improved functional outcome (pooled odds ratio [OR], 2.02; 95% confidence interval [CI], 1.64–2.50), lower mortality (OR, 0.75; 95% CI, 0.58–0.97), and similar symptomatic intracranial hemorrhage rate (OR, 1.12; 95% CI, 0.72–1.76) compared with standard medical care. A higher proportion of functional independence was seen in patients with terminus intracranial artery occlusion (±M1) (OR, 3.16; 95% CI, 1.64–6.06), baseline Alberta Stroke Program Early CT score of 8–10 (OR, 2.11; 95% CI, 1.25–3.57) and age ≤70 years (OR, 3.01; 95% CI, 1.73–5.24). EVT performed under conscious sedation had better functional outcomes (OR, 2.08; 95% CI, 1.47–2.96) without increased risk of symptomatic intracranial hemorrhage or short-term mortality compared with general anesthesia.

**Conclusions:**

Vessel-imaging proven large vessel occlusion, a favorable scan, and younger age are useful predictors to identify anterior circulation stroke patients who may benefit from EVT. Conscious sedation is feasible and safe in EVT based on available data. However, firm conclusion on the choice of anesthetic types should be drawn from more appropriate randomized controlled trials.

## Introduction

Endovascular treatment (EVT) with either mechanical devices or intra–arterial thrombolysis to remove or dissolve blood clots has long been regarded as a potent therapy for acute ischemic stroke, especially for patients with intracranial large vessel occlusion (LVO) resistant to intravenous recombinant tissue–type plasminogen activator (rt–PA).[1–3] However, three initial randomized controlled trials (RCTs) failed to show a benefit of EVT compared with intravenous rt–PA, leading to widespread pessimism in the neurological community about the interventional therapy.[4–6] The lack of confirmed intracranial artery occlusions by pretreatment vessel imaging, use of early–generation mechanical devices, delayed treatment initiation, and non–consecutive subject enrollment were considered to contribute significantly to these neutral results.[7] Such profound critiques have facilitated improvement in the study design and methodology used in subsequent trials.

Five published RCTs have shown consistent and persuasive superiority of EVT, predominantly mechanical thrombectomy with use of stent retrievers, in patients with LVO in anterior circulation.[8–12] These studies have been further supported by two more RCTs that reported a positive result or a positive trend after interim analyses.[13,14] On the strength of these positive findings, updated American Heart Association/American Stroke Association guideline recommends mechanical thrombectomy with a stent retriever as class I, level A evidenced–based treatment.[15] A recent meta-analysis concluded that endovascular treatment in addition to intravenous thrombolysis yields improved functional outcome and lower mortality after 3 months compared with intravenous thrombolysis alone.[16] However, this review was not exhaustive and did not examine trials that compared intra-arterial thrombolysis alone or in combination with mechanical thrombectomy with intravenous thrombolysis, nor did it explore possible subgroup effects. Uncertainty and controversy still remain about the impact of age, stroke severity, occlusion site, and anesthetic types on EVT outcomes and quantitative evidence from existing RCTs are lacking. Therefore, we performed this meta–analysis to evaluate the efficacy and safety of EVT overall and in pre-specified subgroups with available data.

## Materials and Methods

### Data Sources and Searches

A comprehensive search for eligible studies was conducted from April 2015 to July 2015 using the following major databases from their earliest inception with no restriction on publication year or language: MEDLINE (via PubMed), EMBASE (via OVID), Cochrane Central Register of Controlled Trials (via OVID), and clinicaltrials.gov (http://www.clinicaltrials.gov). We also searched conference proceedings and reference lists of relevant studies. (S1 File)

### Inclusion and Exclusion Criteria

Inclusion criteria for this study were as follows: (1) multicenter or single–center RCTs with a follow–up period of ≥90 days; (2) receipt of a diagnosis of acute ischemic stroke confirmed by neuroimaging (number of patients ≥10); (3) an intervention involving EVT (mechanical thrombectomy with clot aspiration, coil retrievers or stent retrievers, intra–arterial thrombolysis with rt–PA, or a combination of both; (4) a comparison involving standard medical care according to guidelines from professional medical societies, including intravenous rt–PA in eligible patients; and (5) outcomes for functional independence defined as a modified Rankin scale (mRS) score of 0 to 2, symptomatic intracranial hemorrhage (sICH), and all–cause mortality within 90 days. SICH was defined as any type of intracerebral hemorrhage, or parenchymal hemorrhage type 2 related to clinical deterioration with a four–or–more point increase in the National Institutes of Health Stroke Scale (NIHSS) score, according to criteria of the National Institute of Neurological Disorders and Stroke (NINDS), the European Cooperative Acute Stroke Study (ECASS), the Safe Implementation of Thrombolysis in Stroke–Monitoring Study (SITS–MOST), or the trial’s own definition.[17–19] (S1 Table)

### Study Selection, Data Extraction and Quality Assessment

Two investigators (FO, YC) independently screened all titles and abstracts, obtained and reviewed the full text of selected records, and used a specially designed data extraction form to retrieve data from each eligible study. We extracted the following data from published papers, protocols, appendices, and post hoc analyses: author, year, country, number of patients in total and in each arm, inclusion and exclusion criteria, age, baseline NIHSS score, ASPECTS, occlusion sites, type of EVT, type of anesthesia, and the number of patients in each treatment arm with outcome events. Given that some trials defined a favorable functional outcome as a mRS score of 0 to 1 or as an overall distribution, number of patients in each mRS score category was also sought. Outcome data according to different anesthesia types were only sought for the EVT arm. In the case of missing data, corresponding authors were contacted for additional information, if possible. Any disagreement was resolved by discussion and consultation with a third investigator (YZ) if needed. The data were entered into Cochrane Review Manager software (RevMan, version 5.3) by one investigator (FO) and reviewed by another (YC). The two investigators performed a quality assessment of eligible studies with the Cochrane Risk of Bias Tools.[20] Any disagreement was resolved by consensus.

### Data Synthesis and Analysis

Statistical analysis was performed using RevMan, version 5.3. We used the Mantel–Haenszel method to calculate pooled odds ratios (ORs) and their 95% confidence intervals (CIs) with dichotomous outcomes extracted from individual trials. Heterogeneity among studies was evaluated using the *I*^*2*^ statistic for each outcome and *I*^*2*^ ≥50% was considered as indicating significant heterogeneity. The fixed–effect model was applied in the absence of significant heterogeneity; otherwise, a random–effect model was employed. Statistical significance was considered to be *P* <0.05. We aimed to include all trials in the primary analysis and further restricted the assessment to trials with pretreatment confirmation of intracranial artery occlusion by vessel imaging. We also planned to conduct pre–specified subgroup analyses for patients stratified by occlusion site, baseline ASPECTS, age, baseline NIHSS score, and anesthesia type.

## Results

### Study Characteristics

**Fig 1:**
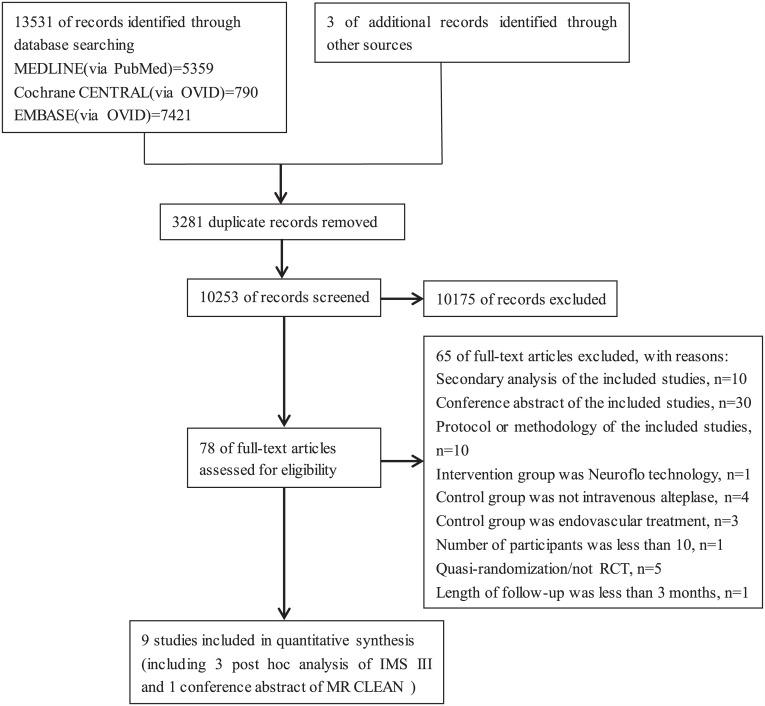
Study selection flow diagram. CENTRAL = Cochrane Central Register of Controlled Trials; IMS III = Interventional Management of Stroke III Study; MR CLEAN = a Multicenter Randomized Clinical trial of Endovascular treatment for Acute ischemic stroke in the Netherlands.

A total of 10253 unique records were identified through the database search; three additional articles were identified from other sources. Nine trials (SYNTHESIS[21], SYNTHESIS EXPANSION[4], IMS III[5], MR RESCUE[6], MR CLEAN[8], EXTEND-IA[9], ESCAPE[10], SWIFT PRIME[11], and REVASCAT[12]) with a total of 2476 patients were included in the final analysis. Three post hoc analyses of the IMS III trial [22–24] and one abstract of the MR CLEAN trial presented at the 2015 International Stroke Conference[25] were also included (Fig 1). The sample size of each trial ranged from 54 to 656. Table 1 and S2 File provide details about the study characteristics of each trial.

**Table 1:** Summary of the characteristics of included studies.

	Ciccone 2010[21]	Ciccone 2013[4]	Broderick 2013[5]	Kidwell 2013[6]	Berkhemer 2015[8]	Campbell 2015[9]	Goyal 2015[10]	Saver 2015[11]	Jovin 2015[12]
No.of centers (country)	4(Italy)	24(Italy)	58(41US,7Canada,4Australia,6Europe)	22(21US,1Canada)	16(Netherlands)	10(9Australia,1New Zealand)	22(11Canada,6US,3South Korea,1Ireland, 1UK)	39(24US,15Europe)	4(Spain)
No.of participants	54	362	656	118	500	70	315	196	206
Intervention	IAT (25)	IAT (181)	EVT(434)	EVT ± IVT(64)	EVT ± IVT(233)	EVT + IVT (35)	EVT ± IVT(165)	EVT + IVT(98)	EVT ± IVT(103)
Control	IVT(29)	IVT(181)	IVT(222)	Standard medical care ± IVT(54)	Standard medical care ± IVT(267)	IVT(35)	Standard medical care ± IVT (150)	IVT(98)	Standard medical care ± IVT(103)
Time to treatment/h									
EVT	6	6	5	8	6	6	12	6	8
IVT	3	4.5	3	4.5	4.5	4.5	4.5	4.5	4.5
Median Age (SD) /yr									
Intervention	60.6 (13.7)	66 (11)	69(23–89) †	66.4(13.2) #	65.8(54.7–76.0)	68.2(12.3)	71(61–81)	65.0(12.5)	65.7(11.3)
				65.8(16.9) * *					
Control	64.0 (11.7)	67(11)	68(23–84) †	61.6(12.0) #	65.7(55.5–76.4)	70.2(11.8)	70(60–80)	66.3(11.3)	67.2(9.5)
				69.4(15.9) * *					
Median NIHSS score (IQR)*									
Intervention	17(11–19)	13(9–17)	17(7–40) †	16(12–18) #	17(14–21)	17(9–19)	16(13–20)	17(13–20)	17(14–20)
				19(17–22) * *					
Control	16(12–19)	13(9–18)	16(8–30) †	16(11–18) #	18(14–22)	13(13–20)	17(12–20)	17(13–19)	17(12–19)
				20.5 (17–23) * *					
Median ASPECTS (IQR) ‡									
Intervention	NA	NA	NA	NA	9(7–10)	NA	9(8–10)	9(7–10)	7(6–9)
Control	NA	NA	NA	NA	9(8–10)	NA	9(8–10)	9(8–10)	8(6–9)
Pretreatment vessel imaging	Not used	Not used	CTA/MRA	CTA/MRA	CTA/MRA	CTA/MRA	CTA	CTA/MRA	CTA/MRA
Occlusion sites	Anterior circulation(47)	Anterior circulation(330)	ICA(65)	ICA(20)	ICA(4)	ICA(22)	ICA+M1(84)	ICA(32)	ICA(1)
	Posterior circulation(5)	Posterior circulation(29)	M1(135)	M1(78)	ICA+M1(134)	M1(38)	M1/all M2(216)	M1(134)	ICA+M1(53)
			M2(83)	M2(20)	M1(319)	M2(10)	Single M2(9)	M2(19)	M1(131)
			BA(4)		M2(39)				M2(18)
			Control NA		A1/A2(3)				
Types of EVT	IAT alone(10)	IAT alone(109)	MT alone(68)	MT alone (53)	MT alone(171)	MT alone(28)	MT alone(151)	MT alone (98)	MT alone(98)
	IAT+ MT(9)	IAT+MT(56)	MT+IAT(266)	MT+IAT(8)	MT±IAT(24)				
					IAT alone(1)				
Maximum dosage of IA rt-PA	0.9mg/kg	0.9mg/kg	22mg	14mg	90mg,30mg if IVT was used	NA	NA	NA	NA
Mechanical devices	NA	Merci(5)	Merci(95)	Merci(37),	NA	Solitaire^™^FR (28)	Solitaire^™^FR(100)	Solitaire^™^ FR or Solitaire^™^ 2 (87)	Solitaire^™^ FR(98)
		Penumbra(9)	Penumbra(54)	Penumbra(14)					
		Solitaire^™^FR(18)	Solitaire^™^ FR(5)	Both(10)					
		Trevo(5)							
Stent retrievers %	NA	12.70%	1.20%	0%	81.50%	80.00%	78.80%	88.80%	95.10%
IV rt-PA %									
Intervention	NA	NA	100%	43.80%	87.10%	100%	72.70%	100%	67.90%
Control	96.60%	98.30%	100%	29.60%	90.60%	100%	78.70%	100%	77.70%
General anesthesia in EVT (%)	NA	22(12.2%)	143(32.9%)	NA	88(37.8%)	12(34.3%)	15(9.1%)	36(36.7%)	7(6.8%)
Onset to imaging/min									
Intervention	NA	NA	NA	NA	NA	NA	134	NA	192
Control	NA	NA	NA	NA	NA	NA	136	NA	183
Onset to IVT/min									
Intervention	NA	NA	122.4	NA	85	127	110	110.5	117.5
Control	155	165	121.2	NA	87	145	125	117	105
Onset to randomization/min									
Intervention	125	148	NA	NA	204	NA	169	190.5	223
Control	125	145	NA	NA	196	NA	172	188	226
Onset to groin puncture	NA	NA	NA	NA	332[23]	248	241	252||	355
Onset to reperfusion in EVT	NA	NA	NA	NA	332[23]	248	241	252||	355
Reperfusion%(TICI 2b-3) §	NA	NA	41%	27%	58.70%	86%	72.40%	88%	67%

BA = basilar artery, CTA = Computed tomography angiography; EVT = endovascular treatment; IAT = intra-arterial thrombolysis with rt-PA; ICA = intracranial carotid artery; IQR = interquartile range; IVT = intravenous thrombolysis with rt-PA; M1 = the first segment of middle cerebral artery; M2 = the second segments of middle cerebral artery; MRA = MR angiography; MT = mechanical thrombectomy; NA = not available; No = number; SD = standard deviation; TICI = thrombolysis in cerebral infarction.

* NIHSS: the National Institutes of Health Stroke Scale, a quantitative measure of neurological dysfunction after stroke ranging from 0 to 42, with a higher score indicating more severe neurological deficit;

^†^ Mean (range);

^‡^ ASPECTS: the Alberta Stroke Program Early CT score; a 10-point scoring system to quantify early ischemic changes in the middle cerebral artery territory, with a score of 10 indicating normal and 1 point subtracted for each abnormal region;

^§^ TICI: a scoring system used to measure reperfusion of distal branches of occluded artery after mechanical thrombectomy, with 0 indicating no reperfusion, 1 little or slow distal reperfusion, 2a partial reperfusion of less than half of the distal branches, 2b partial reperfusion of more than half of the distal branches and 3 complete reperfusion. A TICI 2b to 3 is considered as substantial reperfusion;

^||^ Time to the first deployment of stent retrievers;

^#^ Penumbral subgroup;

* * Nonpenumbral subgroup.

### Quality Assessment

The quality of randomization and allocation concealment in the included studies was considered to be adequate, except for one trial (SYNTHESIS) in which the same person prepared the casual numbers as well as allocation envelops. Performance bias was observed across all trials since no sham procedure was performed in the control group–thus participants and personnel were not blinded to the treatment assignment; all trials reported intention–to–treat results; six of the included studies were halted before completion of estimated sample size, which may have led to a lack of statistical power. (S3 File, S1 and S2 Figs)

### Synthesis of Results

#### Primary Analysis

**Fig 2:**
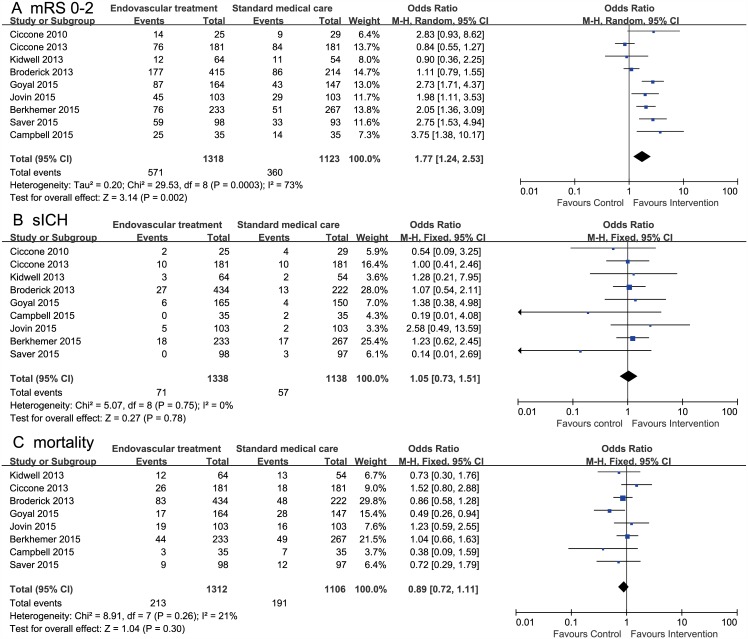
Forest plots of 90-day outcomes between endovascular treatment and standard medical care. **A,** Modified Rankin scale score of 0 to 2. B, Symptomatic intracranial hemorrhage. C, All-cause mortality.

Primary analysis included nine RCTs comparing EVT and standard medical care alone [4–6,8–12,21]. For mRS scores of 0 to 2, data were available for 2441 patients from nine trials; there was a significant effect in favor of EVT (OR, 1.77; 95% CI, 1.24–2.53; *I*^*2*^ = 73%). For sICH within 90 days, data were available for 2476 patients from nine trials; there was no evidence of excess risk for sICH in the EVT group (OR, 1.05; 95% CI, 0.73–1.51, *I*^*2*^ = 0%). For all–cause mortality within 90 days, data were available for 2418 patients from eight trials; there was no difference between the two groups (OR, 0.89; 95% CI, 0.72–1.11; *I*^*2*^ = 0%). (Fig 2)

#### Intracranial LVO Confirmed by Pretreatment Non–invasive Vessel Imaging

**Fig 3:**
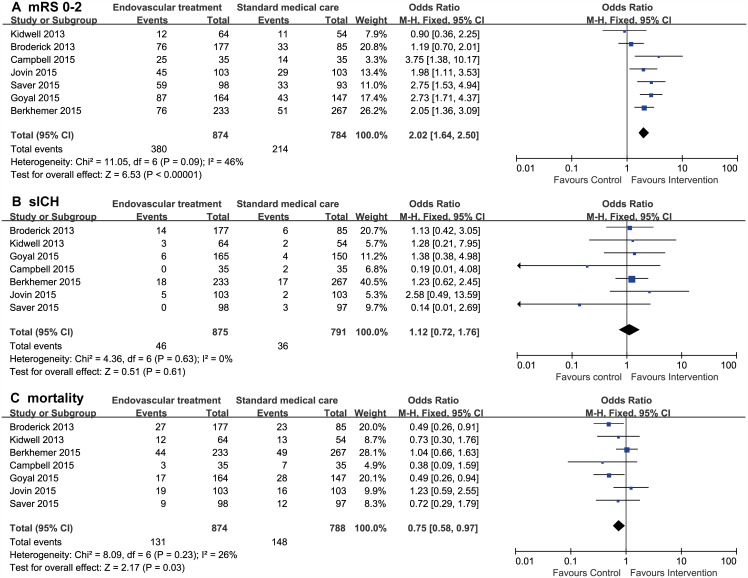
Forest plots of 90-day outcomes between endovascular treatment and standard medical care in patients with proven intracranial artery occlusion. **A,** Modified Rankin scale score of 0 to 2. (B) Symptomatic intracranial hemorrhage. C, All-cause mortality.

Seven trials, including a post hoc analysis of the IMS III trial, reported all outcomes of interest in a subpopulation with intracranial LVO confirmed by pretreatment vessel imaging including computed tomography angiography (CTA) or magnetic resonance angiography (MRA)[6,8–12,22]. Compared with standard medical care alone, EVT had a significantly higher proportion of functional independence (OR, 2.02; 95% CI, 1.64–2.50), lower mortality within 90 days (OR, 0.75; 95% CI, 0.58–0.97), and similar sICH rate (OR, 1.12; 95%CI, 0.72–1.76). No significant heterogeneity among studies was noted with a value of *I*^*2*^ statistic ranging from 0% to 46% for each outcome. (Fig 3)

#### Occlusion Site

**Fig 4:**
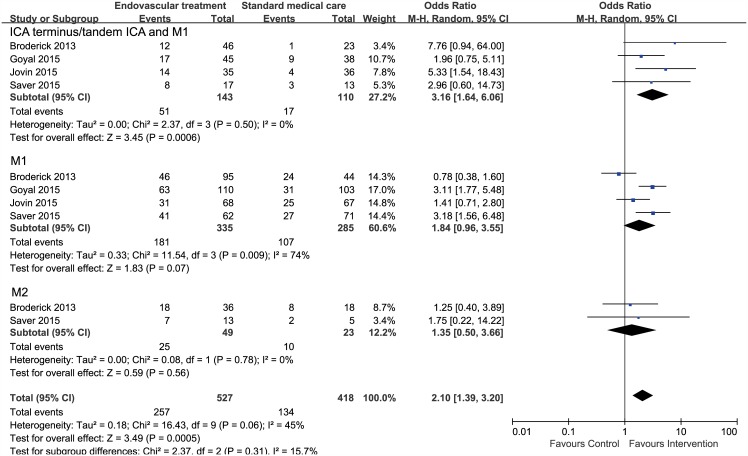
Forest plots of 90-day functional independence between endovascular treatment and standard medical care in patients stratified by occlusion site. ICA = intracranial carotid artery, M1 = the first segment of middle cerebral artery, M2 = the second segments of middle cerebral artery.

Only functional outcome for patients stratified by occlusion site was available from a post hoc analysis of the IMS III trial[22] and pre–specified subgroup analyses of three trials (ESCAPE[10], SWIFT PRIME[11], and REVASCAT[12]). For intracranial carotid artery (ICA) occlusion or tandem occlusion of ICA and the first segment of the middle cerebral artery (M1), data were available for a total of 253 patients; there was a significant effect in favor of EVT (OR, 3.16; 95% CI, 1.64–6.06; *I*^*2*^ = 0%). For M1 occlusion, data from four studies with a total of 620 patients were available; there was a trend toward more favorable outcomes in the intervention group (OR, 1.84; 95% CI, 0.96–3.55; *I*^*2*^ = 74%), though not statistically significant. Only two studies with a relatively small sample size of 72 patients reported functional outcomes for M2 occlusion; pooled analyses revealed no significant difference in functional outcome between two groups (OR, 1.35; 95% CI, 0.50–3.66; *I*^*2*^ = 0%). (Fig 4)

#### Baseline ASPECTS

**Fig 5:**
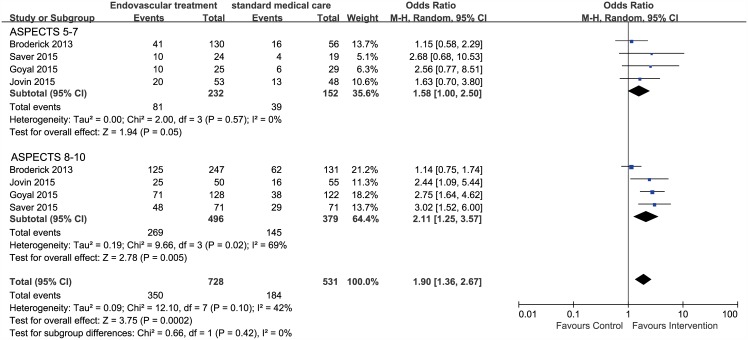
Forest plots of 90-day functional independence between endovascular treatment and standard medical care in patients stratified by baseline Alberta Stroke Program Early CT score (ASPECTS).

Only functional outcome in patients stratified by ASPECTS was reported in a post hoc analysis of the IMS III trial[23] and pre–specified subgroup analyses of three trials (ESCAPE[10], SWIFT PRIME[11], and REVASCAT[12]). Our analyses demonstrated a positive association between favorable scan (ASPECTS 8 to 10) and favorable functional outcome (OR, 2.11; 95% CI, 1.25–3.57, *I*^*2*^ = 69%). In patients with ASPECTS 5 to 7, a higher proportion of functional independence was observed, but the effect estimate just reached statistical significance (OR, 1.58; 95% CI, 1.00–2.50, *I*^*2*^ = 0%). (Fig 5)

#### Age

Only data of functional dependence were available from two trials (SWIFT PRIME[11]and REVASCAT[12]). Patients aged less than 70 years clearly benefited from EVT (OR, 3.01; 95% CI, 1.73–5.24; *I*^*2*^ = 0%), but elderly patients (age ≥70) did not benefit from EVT (OR, 1.50; 95% CI, 0.48–4.70; *I*^*2*^ = 69%). (S3 Fig)

#### Baseline NIHSS Score

Only data of functional outcome were available from two trials (IMS III[4] and ESCAPE[10]). The subgroup analysis revealed a trend toward better outcome in patients with more severe stroke (NIHSS ≥20) (OR, 2.32; 95% CI, 0.90–5.96; *I*^*2*^ = 54%). (S4 Fig)

#### Anesthetic Type

Data of functional outcome, sICH, and all–cause mortality at seven days or at discharge were available from two trials (IMS III[24] and MR CLEAN[25]). Compared with general anesthesia, EVT performed under conscious sedation yielded better functional outcome (OR, 2.08; 95% CI, 1.47–2.96; *I*^*2*^ = 0%) without increased risk of sICH (OR, 0.73; 95% CI, 0.39–1.37; *I*^*2*^ = 0%) or short–term mortality (OR, 0.46; 95% CI, 0.15–1.42; *I*^*2*^ = 81%). (S5 Fig)

### Publication Bias

Publication bias could not be assessed due to the small number of included trials (<10).[26]

## Discussion

Our meta–analysis demonstrated that EVT improved functional outcome and reduced mortality within 90 days for acute ischemic stroke patients with confirmed intracranial LVO in anterior circulation compared with those receiving standard medical care alone. Treatment effect existed largely for patients with ICA or tandem ICA and M1 occlusions, a favorable scan (ASPECTS 8–10), younger age (<70 years), or receiving EVT under conscious sedation.

Confirmation of intracranial artery occlusion with non–invasive vessel imaging prior to treatment is useful for identifying patients who are more likely to benefit from EVT. Pretreatment vessel imaging using CTA or MRA to document treatable occlusions was not required during the SYNTHESIS or SYNTHESIS Expansion trial, and was not a routine practice until the later phase of the IMS III trials. At the beginning of the IMS III trial, a baseline NIHSS score of ≥10 was used to identify patients who might be more likely to experience major arterial occlusion on the basis of previous studies that suggested a significant association between NIHSS score and the presence and location of a vessel occlusion.[27,28] Our analysis regarding baseline stroke severity also found a non–significant trend toward benefit of EVT among patients with a higher NIHSS score (≥20), which may indicate more proximal artery occlusions. However, 80 of 423 patients in the IMS III trial randomized to the intervention group did not receive treatment due to absence of target occlusions. The effect magnitude of EVT might be underappreciated when intention–to–treat analysis was performed based on these data. Therefore, baseline NIHSS score cannot substitute for vessel imaging in terms of sensitivity and specificity in predicting large artery occlusions to select candidates for EVT.[29] Apart from identifying the size and location of occlusions, pretreatment vessel imaging provides information about the status of the aortic arch, extra–cranial vessels, and the circle of Willis, which helps interventionists choose the appropriate device and plan an optimal procedure, and in turn may help to reduce time to treatment.[30] Due to limited data, we failed to further compare the impact of pretreatment CTA and MRA. Further analysis by occlusion site showed that patients with terminus ICA or combined ICA and M1 occlusions benefited most from EVT. The likelihood of an improved outcome was demonstrated as well in patients with M1 occlusion. Our results consolidated the superiority of EVT in opening proximal artery occlusions that might otherwise risk a low rate of reperfusion and subsequent poor clinical outcomes when treated with intravenous rt–PA alone. Data for M2 occlusion revealed no difference between the two reperfusion therapy in 90–day functional outcomes, but it was not conclusive as the statistical power was limited by a small sample size. It is worth noting that a recent case series using stent retrievers indicated the feasibility and safety of EVT in M2 occlusion, especially for patient with moderate to severe neurological deficits.[31,32] These findings require confirmation in large–scale RCTs. In addition, all published RCTs enrolled few patients with acute basilar artery occlusion; the assumption that EVT is superior to intravenous rt–PA for basilar artery occlusion has been challenged by the results of early case series and the observational Basilar Artery International Cooperation study registry (BASIC).[33,34] The results of two ongoing RCTs evaluating the efficacy and safety of EVT for basilar artery occlusion, with a therapeutic time window up to six hours and eight hours respectively, are anticipated to give some insight into this issue.[35,36]

Evaluation of early ischemic change using ASPECTS with non–contrast computed tomography (NCCT) might also ease the task of patient selection. At present, NCCT is still the modality of choice to image stroke patients in the majority of medical centers worldwide due to its quick acquisition and wide availability. The predictive value of ASPECTS in stroke outcomes after reperfusion therapy has been discussed before with inconclusive result.[37–39] In our pooled analysis, among patients with proven artery occlusion, a favorable scan (ASPECTS 8–10) conferred an approximately two–fold or greater chance of 90-day functional independence. Remarkably, our results suggested that patients with less favorable scans (ASPECTS 5–7) can also benefit, although the effect size was close to unity. Since the majority of trials in this meta–analysis excluded patients with baseline ASPECTS of <5, which indicates the greatest burden of ischemic change, data concerning this subgroup was lacking. A threshold of ASPECTS <5 for excluding patients would be reasonable, as it has been suggested that very low ASPECTS identify a subgroup with dismal outcomes and a high rate of sICH after EVT.[40,41] However, the limitation of this grading system cannot be ignored. That is, it has only moderate inter–rater reliability in the ultra–acute phase (within 90 minutes) after stroke onset, and is restricted to middle cerebral artery territory.[42] Moreover, it is based on a snapshot of ischemic change in the brain, and thus its predicted value would be weakened by prolonged time from imaging to treatment.[30] In addition to ASPECTS, some trials used more selective criteria that included a small infarct core and a substantial amount of salvageable penumbra identified by more advanced imaging techniques (e.g., computed tomography perfusion) to exclude patients with extensive ischemic damage.[9,11] Specific imaging–based criteria with good reliability and wide availability in clinical settings must be validated by future research.

Selecting patients by age has not been verified based on the limited data from two studies. Our results demonstrated a non–significant trend in improved functional outcome in the elderly (age ≥70). However, the two trials included in our analysis applied an upper age limit for patient enrollment (SWIFT PRIME = 18–80 years, REVASCAT = 18–85 years), and thus did not well represent the trend toward the overall cohort of very elderly patient. In the MR CLEAN and ESCAPE trials, where no upper age limit was set, a shift toward better outcome in favor of EVT was also observed for patients ≥80 years. Data regarding safety outcomes were insufficient for this group, except for the ESCAPE trial where a reduced mortality with EVT was reported. [43] Based on the available data, we boldly assume that, as is the case for patients with intravenous rt–PA, the elderly may gain benefit if EVT is initiated as early as possible.[44] Nevertheless, results should be evaluated with caution and safety outcomes should be taken into account in further studies.[45]

In keeping with findings from previous retrospective studies [46–48], our results suggested that patients undergoing general anesthesia had worse functional outcomes compared with those undergoing conscious sedation. Our results also indicate that the sICH rate was equivalent or even lower with conscious sedation versus general anesthesia; in other words, conscious sedation not only feasible but also safe. Many factors are likely to contribute to the negative effects of general anesthesia. For example, compared with conscious sedation, general anesthesia confers a higher risk of hypotension and subsequent decreased cerebral perfusion that may exacerbate ischemic damage.[49] The time needed for induction of general anesthesia may delay treatment initiation, as already indicated in the MR CLEAN trial.[25,49] Furthermore, we fail to monitor neurologic deterioration and make any necessary adjustment during the procedure.[24,49] Nevertheless, the finding of the advantage of conscious sedation over general anesthesia in EVT for acute ischemic stroke is subjected to a lot of bias. First, the data was restricted to a small sample size, and half of them came from IMS III trial where the presence of a large vessel occlusion confirmed by neuroimaging was not required at the early phase; randomization in the two included trials was not done according to anesthesia and choice of anesthesia was primarily at the discretion of local interventionists, which led to potential selection bias. Second, there is no adjustment for important confounding factors (i.e., stroke severity and time to treatment). Usually, patients medically indicated for general anesthesia tend to have more severe stroke or other comorbidities that may negatively affect outcomes, which may have confounded the comparison to some extent. In the IMS III trials, median baseline NIHSS score was higher in the general anesthesia group versus the conscious sedation group (18 versus 16). General anesthesia is still the preferred choice of many interventionists for safety concerns, as it reduces the risk of device–related vessel wall perforation and protects the airway, especially in uncooperative patients.[49] Thus, it is agreed that ultimately choice of anesthetic type should be based on a thorough evaluation of individual patients that considers the potential harms and benefits of each approach.[50] Currently, three prospective RCTs to assess the effect of anesthesia type on EVT outcomes are recruiting patients which may shed light on this issue.[51–53] More comprehensive studies to address the underlying mechanisms of anesthesia are needed as well.

Our findings provided quantitative evidence on overall and subgroup effects of endovascular treatment, based mostly on pre-specified subgroup analyses of each trial where the subgroup variables analyzed, except anesthetic types, were stratification factors at randomization. The subgroup effects were also supported by evidence from previous related studies. However, small sample size, potential imbalance in baseline characteristics, and lack of individual data have weakened the claimed credibility of subgroup effects.[54] We failed to evaluate the safety outcomes of these pre–specified subgroups and the impact of M2 or more distal occlusion, basilar artery occlusion, older age, and milder or more sever stroke on EVT outcomes. We also failed to perform a separate and thorough analysis of IA rt-PA for the following reasons. First, of the nine randomized controlled trials included, only two trials (SYNTHESIS and SYNTHESIS EXPANSION) provided data specific to IA rt-PA and data of subgroup analysis from these two trials were not available. Second, the dose of IA rt-PA was not established and varied among studies, which made it difficult to combine and compare. Third, there were different reperfusion strategies including IA rt-PA alone, combined IA rt-PA + IV rt-PA, combined IA rt-PA + mechanical thrombectomy, or combined IA rt-PA+ mechanical thrombectomy + IV rt-PA. Unfortunately, data were lacking for exploring potential differences in the safety and efficacy of these individual methods. Future inclusive and well-design trials are expected to resolve these uncertainties.

## Conclusion

In conclusion, this meta–analysis of all prospective RCTs demonstrated the overall superiority of EVT in improving clinical outcomes for acute stroke patients compared with standard medical care alone. The treatment effect was more robust for patients with confirmed LVO, ICA±M1 occlusions, higher ASPECTS, and younger age. Although EVT performed under conscious sedation was associated better functional outcome versus general anesthesia, the conclusion cannot be drawn on the choice of anesthetic types in EVT for acute ischemic stroke from limited data. A retrospective patient–level pooled analysis of all endovascular trials and more prospective RCTs are anticipated in the near future to make a more precise assessment of EVT effect in different subsets of patients. [55]

## Supporting Information

S1 Fig Risk of bias summary. (EPS)

S2 Fig Risk of bias graph. (EPS)

S3 Fig Forest plot of 90-day functional independence between endovascular treatment and standard medical care in subgroups stratified by age. (PDF)

S4 Fig Forest Plot of 90-day functional independence between endovascular treatment and standard medical care in subgroups stratified by the National Institutes of Health Stroke Scale (NIHSS) score. (PDF)

S5 Fig Forest plots of 90-day outcomes between endovascular treatment and standard medical care in patients stratified by anesthetic type. CS = conscious sedation, GA = general anesthesia. (A) Modified Rankin scale score of 0 to 2. (B) Symptomatic intracranial hemorrhage. (C) All-cause mortality.
(EPS)

S1 File Search Strategy. (DOCX)

S2 File Quality Assessment. (DOCX)

S3 File Study Characteristics. (DOCX)

S4 File The PRISMA Checklist of the Manuscript. (DOC)

S5 File The Data Set. (RM5)

S1 Table Definitions of Symptomatic Intracranial Hemorrhage. (DOCX)
