# Supplementary material for: Selection of Patients and Anesthetic Types for Endovascular Treatment in Acute Ischemic Stroke: A Meta-Analysis of Randomized Controlled Trials
Source: PLoS One. 2016 Mar 8;11(3):e0151210. doi: 10.1371/journal.pone.0151210 (PMC4783038; doi:10.1371/journal.pone.0151210)
Supplement: S3 Fig — (PDF) [file pone.0151210.s003.pdf]

Study or Subgroup	Experimental Events	Experimental Total	Control Events	Control Total	Weight	Odds Ratio M-H, Random, 95% CI
age <70						
Jovin 2015	32	61	14	60	26.8%	3.63 [1.66, 7.92]
Saver 2015	36	55	22	51	26.7%	2.50 [1.14, 5.47]
**Subtotal (95% CI)**		**116**		**111**	**53.5%**	**3.01 [1.73, 5.24]**
Total events	68		36			
Heterogeneity: Tau² = 0.00; Chi² = 0.44, df = 1 (P = 0.51); I² = 0%						
Test for overall effect: Z = 3.90 (P < 0.0001)						
age ≥70						
Saver 2015	23	43	12	40	23.3%	2.68 [1.09, 6.62]
Jovin 2015	13	42	15	43	23.2%	0.84 [0.34, 2.07]
**Subtotal (95% CI)**		**85**		**83**	**46.5%**	**1.50 [0.48, 4.70]**
Total events	36		27			
Heterogeneity: Tau² = 0.47; Chi² = 3.19, df = 1 (P = 0.07); I² = 69%						
Test for overall effect: Z = 0.70 (P = 0.49)						
**Total (95% CI)**		**201**		**194**	**100.0%**	**2.18 [1.19, 3.99]**
Total events	104		63			
Heterogeneity: Tau² = 0.20; Chi² = 6.22, df = 3 (P = 0.10); I² = 52%						
Test for overall effect: Z = 2.52 (P = 0.01)						
Test for subgroup differences: Chi² = 1.16, df = 1 (P = 0.28), I² = 13.8%						

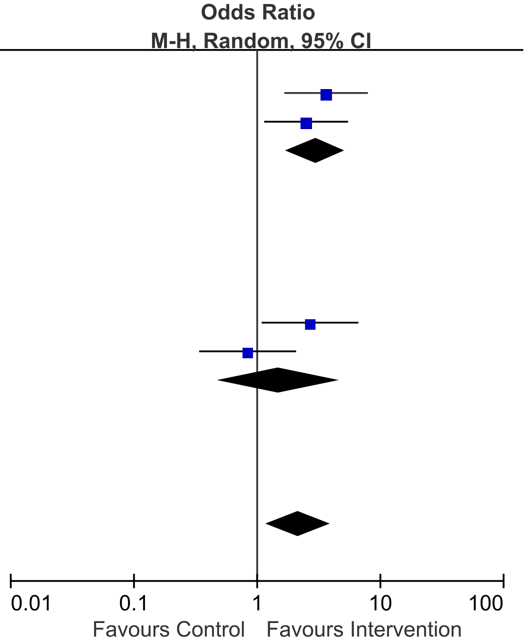

S3 Figure: Forest plots of 90-day functional independence (mRS 0-2) between endovascular treatment and standard medical care in patients stratified by age.
