# Supplementary material for: Selection of Patients and Anesthetic Types for Endovascular Treatment in Acute Ischemic Stroke: A Meta-Analysis of Randomized Controlled Trials
Source: PLoS One. 2016 Mar 8;11(3):e0151210. doi: 10.1371/journal.pone.0151210 (PMC4783038; doi:10.1371/journal.pone.0151210)
Supplement: S4 Fig — (PDF) [file pone.0151210.s004.pdf]

	Endovascular treatment		Standard medical care			Odds Ratio
Study or Subgroup	Events	Total	Events	Total	Weight	M-H, Random, 95% CI
NIHSS≥20						
Broderick 2013	31	130	12	71	23.7%	1.54 [0.73, 3.23]
Goyal 2015	18	43	6	40	17.6%	4.08 [1.42, 11.76]
**Subtotal (95% CI)**		**173**		**111**	**41.3%**	**2.32 [0.90, 5.96]**
Total events	49		18			
Heterogeneity: Tau² = 0.26; Chi² = 2.19, df = 1 (P = 0.14); I² = 54%						
Test for overall effect: Z = 1.75 (P = 0.08)						
NIHSS<20						
Broderick 2013	146	285	74	143	30.8%	0.98 [0.66, 1.46]
Goyal 2015	69	118	37	106	27.9%	2.63 [1.53, 4.51]
**Subtotal (95% CI)**		**403**		**249**	**58.7%**	**1.58 [0.60, 4.14]**
Total events	215		111			
Heterogeneity: Tau² = 0.43; Chi² = 8.21, df = 1 (P = 0.004); I² = 88%						
Test for overall effect: Z = 0.92 (P = 0.36)						
**Total (95% CI)**		**576**		**360**	**100.0%**	**1.85 [0.99, 3.46]**
Total events	264		129			
Heterogeneity: Tau² = 0.29; Chi² = 11.85, df = 3 (P = 0.008); I² = 75%						
Test for overall effect: Z = 1.91 (P = 0.06)						
Test for subgroup differences: Chi² = 0.32, df = 1 (P = 0.57), I² = 0%						

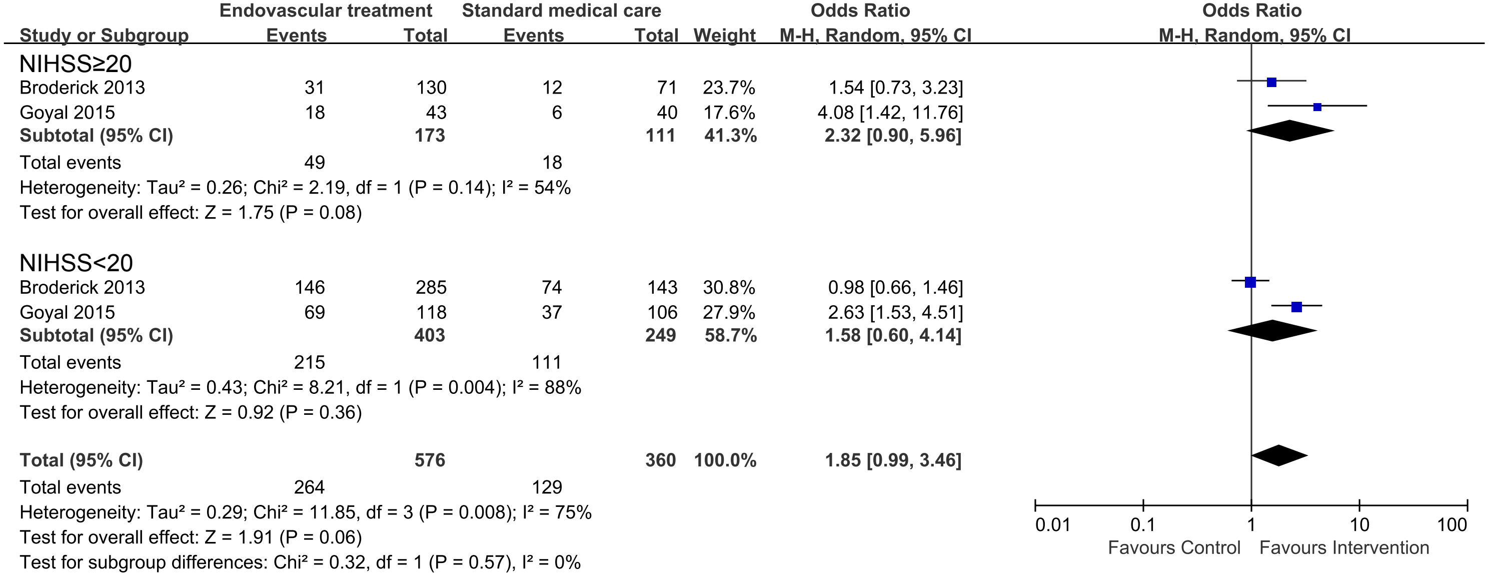

S4 Figure: Forest plots of 90-day functional independence (mRS 0-2) between endovascular treatment and standard medical care in patients stratified by the National Institutes of Health Stroke Scale (NIHSS) score.
