# Supplementary material for: Selection of Patients and Anesthetic Types for Endovascular Treatment in Acute Ischemic Stroke: A Meta-Analysis of Randomized Controlled Trials
Source: PLoS One. 2016 Mar 8;11(3):e0151210. doi: 10.1371/journal.pone.0151210 (PMC4783038; doi:10.1371/journal.pone.0151210)
Supplement: S1 File — (DOCX) [file pone.0151210.s006.docx]

**S File 1: Search Strategy**

The following search strategy, using a combination of MeSH terms and free text terms, was used for MEDLINE(via PubMed) and modified for the other electronic database searches.

**MEDLINE Search Strategy (via PubMed)**

1. brain ischemia[MeSH Terms] or stroke[MeSH Terms] or Intracranial Embolism and Thrombosis[MeSH Terms]
2. (ischemi* and (stroke* or apoplexy* or cerebral vasc* or cerebrovasc* or cva)).tw.
3. ((brain or cerebr* or cerebell* or vertebrobasil* or hemispher* or intracran* or intracerebral or infratentorial or supratentorial or middle cerebr* or mca* or anterior circulation or posterior circulation) and (ischemi* or infarct* or thrombo* or emboli* or occlus* or hypoxi*)).tw.
4. #1 or #2 or #3
5. cerebral revascularization[MH] or reperfusion[MH] or Dilatation[MH] or thrombectomy[MH] or Embolectomy[MH] or Catheterization[MH] or Angioplasty[MH]
6. endovascular procedures[MH] or radiography, interventional[MH] or radiology, interventional[MH] or stents[MH] or catheters,indwelling[MH]
7. (mechanical thrombol* or endovascular device or thromboaspiration or embolectomy or thrombectomy or recanalization).tw.
8. (angioplasty* or stent*).tw.
9. ((clot or thrombus or thrombi or embol*) and (aspirat* or remov* or retriev* or fragmentation or retract* or extract* or obliterate* or dispers*)).tw.
10. ((retrieval or extraction) and device*).tw.
11. Or/5-10
12. thrombolytic therapy[MH] or fibrinolysis[MH] or fibrinolytic agents[MH] or fibrinolysin [MH] or plasminogen activators[MH]
13. (thromboly* or fibrinoly* or recanalis* or recanaliz*).tw.
14. ((clot* or thrombus) and (lyse or lysis or dissolve* or dissolution)).tw.
15. (tPA or t-PA or rtPA or rt-PA or plasminogen or plasmin or alteplase or actilyse).tw.
16. (anistreplase or streptodornase or streptokinase or urokinase or pro?urokinase or rpro?uk or reteplase or tenecteplase).tw.
17. or#12-16
18. Infusion,intra-arterial or injection,intra-arterial[MH]
19. Intra?arterial or intraarterial or IA tw.
20. #18 or #19
21. #17 and #20
22. Randomized Controlled Trials as Topic[MH]
23. random allocation[MH]
24. Controlled clinical trials as topic[MH]
25. control groups[MH]
26. Double-Blind Method[MH] or Single-Blind Method[MH]
27. placebos[MH]
28. randomized controlled trial.pt.
29. controlled clinical trial.pt.
30. random*.tw.
31. placebo* or sham*.tw.
32. controlled and (trial* or stud*).tw.
33. group* .tw.
34. ((singl* or doubl* or tripl* or trebl*) and (blind* or mask*)).tw.
35. or #22-34
36. Animals[MH] not humans[MH]
37. #35 not #36
38. (#4 or #11) and #21 and #37

**EMBASE Search Strategy (via OVID)**

1. exp brain ischemia/ or exp brain infarction/ or exp stroke/ or exp brain embolism/
2. (isch?emi$ adj6 (stroke$ or apoplexy$ or cerebral vasc$ or cerebrovasc$ or cva)).ti,ab.tw.
3. ((brain or cerebr$ or cerebell$ or vertebrobasil$ or hemispher$ or intracran$ or intracerebral or infratentorial or supratentorial or middle cerebr$ or mca$ or anterior circulation) adj5 (isch?emi$ or infarct$ or thrombo$ or emboli$ or occlus$ or hypoxi$)).ti,ab.tw.
4. Or/1-3
5. exp(cerebral revascularization or reperfusion or Dilatation or thrombectomy or Embolectomy or Catheterization or Angioplasty)/
6. exp(endovascular procedures or radiography, interventional or radiology, interventional or stents or catheters,indwelling)/
7. (mechanical thrombol$ or endovascular device or thromboaspiration or embolectomy or thrombectomy or recanalization).tw.
8. (angioplasty$ or stent$).tw.
9. ((clot or thrombus or thrombi or embol$) adj5 (aspirat$ or remov$ or retriev$ or fragmentation or retract$ or extract$ or obliterate$ or dispers$)).tw.
10. ((retrieval or extraction) adj5 device$).tw.
11. or/5-10
12. exp(thrombolytic therapy or fibrinolysis or fibrinolytic agents or fibrinolysin or plasminogen activators)/
13. (thromboly$ or fibrinoly$ or recanalis$ or recanaliz$).tw.
14. ((clot$ or thrombus) adj4 (lyse or lysis or dissolve$ or dissolution)).tw.
15. (tPA or t-PA or rtPA or rt-PA or plasminogen or plasmin or alteplase or actilyse).tw.
16. (anistreplase or streptodornase or streptokinase or urokinase or pro?urokinase or rpro?uk or or reteplase or tenecteplase\).tw.
17. Or/5-16
18. exp intraarterial drug administration/ sh.
19. Intra?arterial or intra arterial or IA).tw.
20. 18 or 19
21. 17 and 20
22. exp Randomized Controlled Trials/as topic
23. exp random allocation/
24. exp Controlled clinical trials/
25. exp control goups/
26. exp(Double-Blind procedure or Single-Blind procedure)/double blind method or sinfl
27. exp placebos/
28. Random$.tw.
29. Placebo$ or sham$.tw.
30. controlled adj 6(trial$ or stud$).tw.
31. group$ .tw.
32. ((singl$ or doubl$ or tripl$ or trebl$) near (blind$ or mask$)).tw.
33. or/22-32
34. exp animals/not exp humans/
35. 33 not 34
36. 4 and (11 or 21) and 35

**Cochrane Central Register of Controlled Trials (via OVID)**

1. exp brain ischemia/ or exp stroke
2. (isch?emi$ adj6 (stroke$ or apoplexy$ or cerebral vasc$ or cerebrovasc$ or cva)).ti,ab,kw.
3. ((brain or cerebr$ or cerebell$ or vertebrobasil$ or hemispher$ or intracran$ or intracerebral or infratentorial or supratentorial or middle cerebr$ or mca$ or anterior circulation) adj5 (isch?emi$ or infarct$ or thrombo$ or emboli$ or occlus$ or hypoxi$)).ti,ab,kw.
4. Or/1-3
5. exp (cerebral revascularization or reperfusion or Dilatation or thrombectomy or Embolectomy or Catheterization or Angioplasty)/
6. exp(endovascular procedures or radiography, interventional or radiology, interventional or stents or catheters,indwelling)/
7. (mechanical thrombol$ or endovascular device or thromboaspiration or embolectomy or thrombectomy or recanalization).ti,ab,kw.
8. (angioplasty$ or stent$).ti,ab,kw.
9. ((clot or thrombus or thrombi or embol$) adj5 (aspirat$ or remov$ or retriev$ or fragmentation or retract$ or extract$ or obliterate$ or dispers$)).ti,ab,kw.
10. ((retrieval or extraction) adj5 device$).ti,ab,kw.
11. Or/5-10
12. exp(thrombolytic therapy or fibrinolysis or fibrinolytic agents or fibrinolysin or plasminogen activators)/
13. (thromboly$ or fibrinoly$ or recanalis$ or recanaliz$).ti,ab,kw.
14. ((clot$ or thrombus) near (lyse or lysis or dissolve$ or dissolution)).ti,ab,kw.
15. (tPA or t-PA or rtPA or rt-PA or plasminogen or plasmin or alteplase or actilyse).ti,ab,kw.
16. (anistreplase or streptodornase or streptokinase or urokinase or pro?urokinase or rpro?uk or or reteplase or tenecteplase).ti,ab,kw.
17. Or/12-16
18. exp(infusions,intra-arterial or injections,intra-arterial)/
19. Intra?arterial or intra arterial or IA).ti,ab,kw.
20. 18 or 19
21. 17 and 20

(11 or 21) and 4
