# Supplementary material for: Selection of Patients and Anesthetic Types for Endovascular Treatment in Acute Ischemic Stroke: A Meta-Analysis of Randomized Controlled Trials
Source: PLoS One. 2016 Mar 8;11(3):e0151210. doi: 10.1371/journal.pone.0151210 (PMC4783038; doi:10.1371/journal.pone.0151210)
Supplement: S2 File — (DOCX) [file pone.0151210.s007.docx]

**S File 2: Quality Assessment of Each Included Study**

**Ciccone 2010**

| **Bias** | **Author’s judgement** | **Support for judgement** |
| --- | --- | --- |
| Random sequence generation  (selection bias) | Unclear risk | Random assignment to treatment was stratified per center and prepared in a ratio 1:1 with casual numbers by a person not involved in the recruitment and not operating in any of the recruiting centers, but great heterogeneity existed in the randomization rate among the four centers. |
| Allocation concealment  (selection bias) | Unclear risk | The same person who prepared the casual numbers prepared opaque, sealed envelopes that were sequentially opened by the randomizing investigators. |
| Blinding of participants and personnel  (performance bias) | High risk | Participants and personnel were not blinded to the treatment assignment. |
| Blinding of outcome assessment  (detection bias) | Low risk | The mRS score at 90 days was evaluated by telephone call by a single trained neurologist blinded to treatment assignment. |
| Incomplete outcome data  (attrition bias) | Low risk | Intention-to-treat results were reported.  Number and reasons for no treatment were presented.  No lost to follow-up. |
| Selective reporting  (reporting bias) | Low risk | All pre-specified outcome results were reported. |
| Other bias | Unclear risk | The trial was stopped early due to upgrade of protocol and reorganization of study, which led to a small sample size compared with the projected number of enrollment.  Data was collected mainly in one of 4 participant centers.  6 out of 25 in the intra-arterial thrombolysis group received no treatment. |

**Ciccone 2013**

| **Bias** | **Author’s judgement** | **Support for judgement** |
| --- | --- | --- |
| Random sequence generation  (selection bias) | Low risk | Randomization was carried out on-line, in a centralized way, after filling out the electronic Case Report Form by local neurologists. |
| Allocation concealment  (selection bias) | Low risk | Central allocation. |
| Blinding of participants and personnel  (performance bias) | High risk | Participants and personnel were not blinded to the treatment assignment. |
| Blinding of outcome assessment  (detection bias) | Low risk | A long-term patient's clinical condition is evaluated by a single neurologist blinded to treatment allocation by telephone interview. |
| Incomplete outcome data  (attrition bias) | Low risk | Intention-to-treat results were reported.  Number and reasons for cross-over were reported.  No lost to follow-up. |
| Selective reporting  (reporting bias) | Low risk | All pre-specified outcomes were reported. |
| Other bias | Unclear risk | Atrial fibrillation was significantly less frequent in the endovascular treatment group.  Dissection as the cause of stroke was more frequent in the endovascular group. |

**Broderick 2013**

| **Bias** | **Author’s**  **judgement** | | **Support for judgement** |
| --- | --- | --- | --- |
| Random sequence generation  (selection bias) | Low risk | Participants were randomly assigned in a 2:1 ratio to endovascular therapy or intravenous t-PA alone with the use of an Internet-based, computerized algorithm of minimization and the biased-coin method. | |
| Allocation concealment  (selection bias) | Low risk | Sealed randomization envelopes placed at each clinical site. A patient was randomized by opening a pre-specified sealed envelope that contained the treatment assignment. The pre-specification of the envelope is done through the web within 8 hours after the previous patient was randomized and his/her enrollment data were entered into the database. | |
| Blinding of participants and personnel  (performance bias) | High risk | Participants and personnel were not blinded to the treatment assignments. | |
| Blinding of outcome assessment  (detection bias) | Low risk | All modified Rankin scores at 90 days were assessed by investigators who were unaware of the treatment assignment.  CT and CTA imaging studies were transferred to the Imaging Analysis Center in Calgary for central interpretation by a blinded three-member consensus panel.  Angiographic data were reviewed by two independent readers, and a third reader provided final adjudication as needed. | |
| Incomplete outcome data  (attrition bias) | Low risk | Intention-to-treat results were presented.  Number and reasons for lost to follow-up were reported.  No cross-over. | |
| Selective reporting  (reporting bias) | Unclear risk | Outcomes of the Barthel Index, Glasgow Outcome Scale, NIHSS, EuroQol EQ-5D and Trail Making Test A and B at 90 days were stated in the protocol of the trial but results were not provided in the full-text paper. | |
| Other bias | Unclear risk | The proportion of patients with a history of coronary artery disease was higher in the standard treatment group.  The proportion of patients with baseline mRS of 2 to 3 were higher in patients randomized to endovascular treatment group. | |

**Kidwell 2013**

| **Bias** | **Author’s judgement** | **Support for judgement** |
| --- | --- | --- |
| Random sequence generation  (selection bias) | Low risk | Imaging data were transferred to a dedicated on-site MR RESCUE computer via local area network for image post-processing. The program automatically analyzed and classified the penumbral pattern in each patient and displayed a 4 digit code for the site to enter into the randomization web site. |
| Allocation concealment  (selection bias) | Low risk | The randomization web site recognized the code as indicating penumbral or non-penumbral pattern and used this information in generating the treatment assignment, with the randomization stratified by penumbral pattern and imaging modality employing a biased coin technique. |
| Blinding of participants and personnel  (performance bias) | High risk | Participants and personnel were not blinded to treatment assignments. |
| Blinding of outcome assessment  (detection bias) | Low risk | The day 90 mRS score was assessed by an investigator blinded to treatment assignment.  Core laboratories completed primary neuroimaging analyses blinded to treatment assignment before database lock. |
| Incomplete outcome data  (attrition bias) | Low risk | Intention-to-treat results were reported.  Number and reasons for drop-out were reported.  No cross-over or lost to follow-up. |
| Selective reporting  (reporting bias) | Low risk | All pre-specified outcomes were reported. |
| Other bias | Unclear risk | The rate of congestive heart failure was significantly lower among patients in the endovascular treatment group.  The median NIHSS score was lower in patients with favorable penumbral pattern in both endovascular and standard treatment groups.  The trial completed over an 8 year period in 22 centers with relatively low rate of recruitment. |

**Berkhemer 2015**

| **Bias** | **Author’s**  **judgement** | | **Support for judgement** |
| --- | --- | --- | --- |
| Random sequence generation  (selection bias) | Low risk | The randomization procedure is computer- and web-based, using permuted blocks. Full-time back-up by telephone is provided. | |
| Allocation concealment  (selection bias) | Low risk | Allocation was full-time back-up by telephone. | |
| Blinding of participants and personnel  (performance bias) | High risk | Participants and personnel were not blinded to the treatment assignments. | |
| Blinding of outcome assessment  (detection bias) | Low risk | The mRS score at 90 days was accessed by a single investigator who was unaware of the treatment assignments. All imaging studies except for DSA were evaluated by two neuro-radiologists who were blinded. Angiographic outcomes on DSA imaging were assessed by an independent core lab. Final infarct volume on the follow-up CT scan was assessed with an automated, validated algorithm. | |
| Incomplete outcome data  (attrition bias) | Low risk | Intention-to-treat results reported.  Number and reasons for drop-out and cross-over were reported.  No lost to follow-up. | |
| Selective reporting  (reporting bias) | Low risk | All pre-specified outcomes were reported. | |
| Other bias | Low risk | The trial included 145 patients with extracranial carotid occlusion, a higher proportion than in any of the other trials. | |

**Campbell 2015**

| **Bias** | **Author’s**  **judgement** | **Support for judgement** |
| --- | --- | --- |
| Random sequence generation  (selection bias) | Low risk | Patients underwent randomization via a centralized website. |
| Allocation concealment  (selection bias) | Low risk | Central allocation. |
| Blinding of participants and personnel  (performance bias) | High risk | Patients and personnel were not blinded to the treatment assignments. |
| Blinding of outcome assessment  (detection bias) | Low risk | Neurological impairment and functional scores were measured by a healthcare professional blinded to the treatment assignment.  Radiological outcome measures were centrally analyzed, blinded to treatment allocation. |
| Incomplete outcome data  (attrition bias) | Low risk | Intention-to-treat results were presented.  Number and reasons for cross-over were reported.  No lost to follow-up |
| Selective reporting  (reporting bias) | Low risk | All pre-specified outcomes were reported. |
| Other bias | Unclear risk | The trial was stopped early due to efficacy. |

**Goyal 2015**

| **Bias** | **Author’s judgement** | **Support for judgement** |
| --- | --- | --- |
| Random sequence generation  (selection bias) | Low risk | Patients were randomized using a real-time, dynamic Internet-based, minimal sufficient balance randomization method. |
| Allocation concealment  (selection bias) | Low risk | Randomization occurred dynamically in real-time by Internet and was fully concealed. |
| Blinding of participants and personnel  (performance bias) | High risk | Participants and personnel were not blinded to the treatment assignment. |
| Blinding of outcome assessment  (detection bias) | Low risk | The mRS score was assessed by trained personnel who were unaware of the treatment assignments. Interpretation of the imaging was performed at an external core laboratory by personnel who were unaware of the treatment-group assignment. |
| Incomplete outcome data  (attrition bias) | Low risk | Intention-to-treat results reported.  Number and reasons for cross-over were reported.  No lost to follow-up. |
| Selective reporting  (reporting bias) | Low risk | All pre-specified outcomes were reported. |
| Other bias | Unclear risk | The trial was stopped early because of efficacy. |

**Saver 2015**

| **Bias** | **Author’s judgement** | **Support for judgement** |
| --- | --- | --- |
| Random sequence generation  (selection bias) | Low risk | Randomization was managed by a third-party vendor which used a minimization algorithm to dynamically assign subjects to treatment arms, implemented using the interactive web/voice response systems. |
| Allocation concealment  (selection bias) | Low risk | Dynamic allocation was used as opposed to more common methods such as block randomization. It was not possible for study sites to anticipate the randomization allocation for upcoming subjects in the trial. |
| Blinding of participants and personnel  (performance bias) | High risk | Participants and personnel were not blinded to the treatment assignment. |
| Blinding of outcome assessment  (detection bias) | Low risk | The 90-day mRS was assessed by study personnel certified in the scoring of the mRS who were blinded to treatment assignment.  Entry and outcome neurovascular images were assessed in a blinded manner by staff at the core imaging lab. |
| Incomplete outcome data  (attrition bias) | Low risk | Intention-to-treat results were reported.  Number and reasons for cross-over were reported. |
| Selective reporting  (reporting bias) | Unclear risk | Outcomes of the Barthel Index, EuroQoL, transient intra-procedural vasospasm stated in the protocol were not reported. |
| Other bias | Unclear risk | The trial was terminated because of efficacy. |

**Jovin 2015**

| **Bias** | **Author’s judgement** | **Support for judgement** |
| --- | --- | --- |
| Random sequence generation  (selection bias) | Low risk | A real-time randomization procedure is implemented via the REVASCAT Trial Website. |
| Allocation concealment  (selection bias) | Low risk | Central allocation. |
| Blinding of participants and personnel  (performance bias) | High risk | Participants and personnel were not blinded to the treatment assignment. |
| Blinding of outcome assessment  (detection bias) | Low risk | Local and external certified assessors who were unaware of study-group assignments separately evaluated the primary outcome variable in each patient by means of a structured interview with video recording.  Neuroimaging results were evaluated by a core lab which was blinded to treatment allocation. |
| Incomplete outcome data  (attrition bias) | Low risk | Intention-to-treat results were reported.  Number and reasons of protocol deviation and drop-out were reported.  No lost to follow-up or cross-over. |
| Selective reporting  (reporting bias) | Low risk | All pre-specified outcomes were reported. |
| Other bias | Unclear risk | The trial was stopped early before formal stopping boundaries were reached after the release of MR CLEAN due to ethical concern. |
