# Supplementary material for: Selection of Patients and Anesthetic Types for Endovascular Treatment in Acute Ischemic Stroke: A Meta-Analysis of Randomized Controlled Trials
Source: PLoS One. 2016 Mar 8;11(3):e0151210. doi: 10.1371/journal.pone.0151210 (PMC4783038; doi:10.1371/journal.pone.0151210)
Supplement: S3 File — (DOCX) [file pone.0151210.s008.docx]

**S File 3: Detailed Characteristics of Each Included Study**

**Ciccone 2010**

| **Methods** | Randomized, multicenter, controlled, blinded end-point, clinical trial. |
| --- | --- |
| **Participants** | Acute ischemic stroke allowing initiation of intravenous rt-PA within 3 hours after symptom onset and intra-arterial treatment within 6 hours from symptom onset.  Age: 18-80.  Baseline NIHSS score: ≤25.  Occlusion sites: Anterior and posterior cerebral circulation.  Subtypes of stroke: Cardiogenic embolism, dissection, large-atherosclerosis, small vessel disease and others. |
| **Intervention** | Intra-arterial thrombolysis with rt-PA (if necessary, associated to or substituted by mechanical clot disruption or retrieval) versus standard dose of intravenous rt-PA.  Intra-arterial thrombolysis should be performed within 1h, the full dose of rt-PA infusion should not exceed 0.9 mg/kg (maximum dose: 90 mg). If a complete recanalization was achieved, rt-PA infusion was interrupted before reaching the maximum dosage. |
| **Outcomes** | Primary outcome: A mRS score of 0 to 1.  Secondary outcome: NIHSS scores at 7 days or at discharge.  Safety outcome:  (1) Death within 7 days.  (2)Symptomatic intracranial hemorrhage within 7 days (defined as any neurologic deterioration due to intracranial hemorrhage) |

**Ciccone 2013**

| **Methods** | Randomized, multicenter, controlled, blinded end-point clinical trial. |
| --- | --- |
| **Participants** | Acute ischemic stroke allowing initiation of intravenous rt-PA within 4.5 hours after symptom onset or the administration of endovascular treatment within 6 hours after symptom onset.  Age: 18-80.  Baseline NIHSS score: No restrict.  Subtypes of stroke: Cardiogenic embolism, dissection, large-artery atherosclerosis, small vessel disease and others. |
| **Intervention** | Endovascular treatment (intra-arterial thrombolysis with rt-PA, mechanical clot disruption or retrieval, or combination of both) versus intravenous rt-PA.  For intra-arterial thrombolysis, full dose of rt-PA was used (0.9mg/kg, maximum dose 90mg). If complete recanalization was achieved, the rt-PA infusion was stopped. In patients with a neurological deficit but no corresponding occlusion, rt-PA was injected to the vascular area presumably affected. |
| **Outcomes** | Primary outcome: Survival free of disability defined as a mRS score of 0 to 1 at 90 days.  Secondary outcome: Mild neurologic deficit defined as NIHSS score ≤6 at 7 days or discharge.  Safety outcome:  (1) Death from any cause at 7 days and 90 days.  (2) Symptomatic intracranial hemorrhage at 7 days.  (3) Neurologic deterioration at 7 days defined as an increase of 4 more points in NIHSS score. |

**Broderick 2013**

| **Methods** | Randomized, multicenter, controlled, blinded end-point, phase III clinical trial. |
| --- | --- |
| **Participants** | Acute ischemic stroke patients who had received intravenous t-PA within 3 hours after symptom onset and had moderate-to-severe neurologic deficit, defined as an NIHSS score≥10, or a score of 8 to 9 with CTA evidence of an occlusion of M1/M2,A1/A2,ICA,BA.  Age: 18-83. |
| **Intervention** | Endovascular treatment (including intra-arterial thrombolysis, mechanical thrombectomy or both) after intravenous rt-PA versus intravenous rt-PA alone.  In amendment 1-4, intravenous rt-PA was stopped at 40mins if a patient was randomized to the endovascular treatment arm. After amendment 5, all patients received the entire standard t-PA dose over an hour.  For intra-arterial thrombolysis, maximum dose of rt-PA was 22mg.  For mechanical thrombectomy, devices including Merci® Retriever, Penumbra System™, Solitaire™ FR were used according to the local interventionist's choice.  Time from symptom onset to groin puncture should be within 5 hours.  Time from symptom onset to reperfusion should be within 7 hours. |
| **Outcomes** | Primary outcome: Functional independence defined as a mRS score of 0-2 at 90 days.  Secondary outcome: Recanalization and reperfusion (TICI 2-3) in the intervention group.  Safety outcome:  (1) All-cause death within 7 days and 90 days.  (2)Symptomatic intracranial hemorrhage within 30 hours, defined as an intracranial hemorrhage related to a decline in neurological status (a 4 or more-point increase in NIHSS score) as well as new or worsening neurologic symptoms. |

**Kidwell 2013**

| **Methods** | Randomized, multicenter, controlled, blinded end-point, phase IIb clinical trial. |
| --- | --- |
| **Participants** | Acute ischemic stroke due to large vessel proximal occlusion in the anterior circulation (ICA/M1/M2) confirmed on neuroimaging within 8 hours after symptom onset. Patients who were treated with intravenous t-PA without successful recanalization were eligible if MRA or CTA after the treatment showed a persistent target occlusion.  Age: 18-85.  Baseline NIHSS score: ≥6, <30.  Patients were randomized stratified according to penumbra patterns on neuroimaging. |
| **Intervention** | Endovascular treatment plus standard medical care versus standard medical care alone.  Any combination of FDA-cleared thrombectomy devices could be used, mainly including the Merci Retriever and Penumbra System. Mechanical thrombectomy procedure should be completed by 9 hours after symptom onset.  Intra-arterial thrombolysis with rt-PA at a dose of less than 14 mg was allowed as rescue therapy within 6 hours after symptom onset. |
| **Outcomes** | Primary outcome: Functional outcome across all mRS ranks at 90 days (shift analysis)  Secondary outcome:  (1) Good functional outcome defined as mRS 0 to 2 at 90 days.  (2) Excellent functional outcome defined as mRS 0 to 1 at 90 days.  (3) Revascularization defined as TICI 2a to 3 at 7 days.  (4) Reperfusion on CT/MRI perfusion imaging defined as a reduction of 90% or more in the volume of the perfusion lesion from baseline with the time until the peak of the residue function of more than 6 seconds at 7 days.  Safety outcome:  (1) All-cause death at 90 days.  (2) Symptomatic intracranial hemorrhage at 24h and 7 days. |

**Berkhemer 2015**

| **Methods** | Randomized, controlled, multicenter, blinded end-point, phase III clinical trial |
| --- | --- |
| **Participants** | Acute ischemic stroke caused by a proximal intracranial arterial occlusion in the anterior circulation confirmed on vessel imaging and could be treated intra-arterially within 6 hours after symptom onset.  Age: ≥18 (no upper limit).  Baseline NIHSS scores ≥2.  Occlusion sites: ICA-T, M1/M2, A1/A2, with or without extra-cranial ICA occlusion or dissection. |
| **Intervention** | Endovascular treatment (including mechanical thrombectomy, intra-arterial thrombolysis, or both) + standard medical care (including intravenous rt-PA in eligible patients) versus standard medical care alone.  For intra-arterial thrombolysis, rt-PA with a maximum dose of 90mg or urokinase with a maximum dose of 1,200,000 IU was allowed. The dose was restricted to 30mg of rt-PA or 400,000 IU of urokinase if intravenous rt-PA was given.  For mechanical thrombectomy, thrombus retraction, aspiration, wire disruption, or retrievable stents were used. |
| **Outcomes** | Primary outcome: The improvement on mRS score at 90 days.(shift analysis)  Secondary outcome:  (1) Favorable functional outcome defined as a mRS score of 0 to 2 and 0 to 1.  (2) NIHSS score at 24 hours, 5 to 7 days or discharge.  (3)The Barthel index, EuroQol Group 5-Dimension Self-Report Questionnaire at 90 days.  (4) Recanalization measured with CTA/MRA at 24 hours. (defined as mAOL 2-3)  (5) Final infarct volume on non-contrast CT at 5 to 7 days.  Safety outcome:  (1)Symptomatic intracranial hemorrhage at 5 to 7 days.(defined as any evidence of intracranial hemorrhage on imaging studies with an increase of 4 or more points in NIHSS score)  (2)All-cause death at 7 days and 30 days. |

**Campbell 2015**

| **Methods** | Randomized, multicenter, controlled, blinded end-point, clinical trial. |
| --- | --- |
| **Participants** | Acute ischemic stroke due to occlusion of ICA or M1/M2 and have salvageable brain tissue and ischemic core of less than 70ml on CT perfusion imaging, with intravenous rt-PA initiated within 4.5 hours after symptom onset. Intra-arterial clot retrieval initiated within 6 hours after symptom onset.  Age: ≥18 (no upper limit).  Baseline NIHSS score: no limit.  Subtypes of stroke: Cardiogenic embolism, large vessel occlusion, others. |
| **Intervention** | Full dose intravenous rt-PA plus mechanical thrombectomy with Solitaire FR versus intravenous rt-PA alone.  Endovascular treatment had to be initiated within 6 hours after symptom onset and completed within 8 hours after onset. |
| **Outcomes** | Primary outcome: Reperfusion at 24 hours( defined as the percentage reduction in the perfusion-lesion volume  between initial imaging and imaging at 24 hours)  and early neurologic improvement(≥8-point reduction on NIHSS or a score of 0-1 at day 3)  Secondary outcome:  (1)Functional outcome measured by mRS scores.(shift analysis)  (2)Reperfusion on perfusion imaging with symptomatic intracranial hemorrhage, ischemic core growth at 24 hours.  Safety outcome:  (1) All-cause death at 90 days.  (2)Symptomatic intracranial hemorrhage within 36 hours, defined as parenchymal hematoma type 2 combined with an increase on the NIHSS of at least 4 points from baseline or the lowest NIHSS value between baseline and 24h. |

**Goyal 2015**

| **Methods** | Randomized, multicenter, controlled, blinded end-point, clinical trial. |
| --- | --- |
| **Participants** | Acute ischemic stroke due to a proximal intracranial occlusion in the anterior circulation (ICA-T/ICA-L/M1/M2）confirmed by CTA or non-contrast CT, with a small infarct core and moderate-to-good collateral circulation within 12 hours after symptom onset (including patients eligible for and treated with intravenous rt-PA and those ineligible for intravenous rt-PA)  Target time from imaging to groin puncture was within 60min and time from imaging to reperfusion within 90 min)  Age: ≥18 years(no upper limit)  Baseline NIHSS score: >5.  ASPECTS: >5. |
| **Intervention** | Mechanical thrombectomy plus standard medical treatment ( including intravenous rt-PA) versus standard medical treatment alone.  Solitaire stent retriever device was recommended. |
| **Outcomes** | Primary outcome: The mRS score at 90 days.(shift analysis)  Secondary outcome:  (1) Recanalization of target vessel occlusion at 24 hours, defined as mAOL 2-3 on CTA.  (2) Reperfusion at the end of endovascular procedure, defined as TICI 2b-3 on DSA.  Safety outcome:  (1) All-cause death within 90 days.  (2)Symptomatic intracranial hemorrhage within 30 hours, defined as intracerebral hemorrhage deemed by the investigator to be the principal cause of neurologic deterioration.  (3) Procedure or device- related complications. |

**Saver 2015**

| **Methods** | Randomized, multicenter, controlled, blinded end-point, clinical trial. |
| --- | --- |
| **Participants** | Acute ischemic stroke due to confirmed intracranial large vessel occlusion (ICA/M1/M2) in the anterior circulation with a target-mismatch penumbra or small-to-moderate infarct core who were receiving or had received intravenous rt-PA within 4.5 hours after symptom onset. Mechanical thrombectomy with the use of a stent retriever should be initiated within 6 hours after symptom onset.  Age: 18-80.  Baseline NIHSS: ≥8, <30.  ASPECTS: >5 |
| **Intervention** | Mechanical thrombectomy after standard dose intravenous rt-PA versus intravenous rt-PA alone in eligible patients.  Solitaire FR and Solitaire 2 devices were the only device allowed in the trial. |
| **Outcomes** | Primary outcome: The mRS score at 90 days.(shift analysis)  Secondary outcome:  (1) Functional independence defined as mRS 0 to 2 at 90 days.  (2) Change in NIHSS score at 27 hours.  (3) Reperfusion defined as TIMI 2b to 3 on DSA at the completion of endovascular procedure.  (4) Successful reperfusion defined as reperfusion of 90% or more of the initial perfusion-lesion volume at 27 hours.  Safety outcome:  (1) All-cause death at 90 days.  (2)Symptomatic intracranial hemorrhage at 27 hours defined as any PH1, PH2, RIH, SAH or IVH associated with a 4 point or more worsening on the NIHSS. |

**Jovin 2015**

| **Methods** | Randomized, multicenter, controlled, blinded end-point, clinical trial. |
| --- | --- |
| **Participants** | Acute ischemic stroke with confirmed proximal anterior circulation occlusion and the absence of a large infarct core on CTA/MRA/DSA within 8 hours after symptom onset, including patients nonresponsive to intravenous rt-PA. In patients nonresponsive to intravenous rt-PA, a post-treatment vessel imaging should be conducted to confirm persistent occlusion before randomization.  Age: 18-85.  Baseline NIHSS score: ≥6.  ASPECTS: ≥7. |
| **Intervention** | Mechanical thrombectomy plus standard medical care (including those nonresponsive to intravenous rt-PA or presented in the late time window) versus standard medical care alone.  For mechanical thrombectomy, only the Solitaire FR device was allowed in the trial. If the Solitaire device failed after a maximum of 6 passes per vessel, no intra-arterial thrombolytic agents or other mechanical rescue therapies were allowed. |
| **Outcomes** | Primary outcome: The mRS scores at 90 days.(shift analysis)  Secondary outcome:  (1) Infarct volumes on CT/MRI at 24 hours.  (2) Vessel revascularization on CTA/MRI at 24 hours.  (3) Dramatic response to treatment at 24 hours defined as a decrease in the NIHSS score of ≥8 from baseline or an NIHSS score of 0 to 2.  (4) NIHSS, BI, EQ-5D scores at 90 days.  (5) Reperfusion defined as TICI 2b to 3 at the end of endovascular procedure.  Safety outcome:  (1) All-cause death at 90 days.  (2)Symptomatic intracranial hemorrhage within 90 days according to SITS-MOST criteria or ECASS II criteria.  (3) Procedure or device-related complications. |
