# Supplementary material for: Selection of Patients and Anesthetic Types for Endovascular Treatment in Acute Ischemic Stroke: A Meta-Analysis of Randomized Controlled Trials
Source: PLoS One. 2016 Mar 8;11(3):e0151210. doi: 10.1371/journal.pone.0151210 (PMC4783038; doi:10.1371/journal.pone.0151210)
Supplement: S1 Table — (DOCX) [file pone.0151210.s011.docx]

**S1 Table: Definitions of symptomatic intracranial hemorrhage**

|  | **Definition** | **Measure timepoint** |
| --- | --- | --- |
| **Ciccone 2009** | Any neurologic deterioration due to ICH.  Neurological deterioration: defined as any major change in the level of consciousness and/or any substantial change in degree of motor deficit and/or new deficits that were clinically significant and persistent or that led to death within 7 days. | Within 7 days  Preferably on day 2 |
| **Ciccone 2013** | Sudden neurological worsening after a period of stable condition or recovery, with documented intracranial hemorrhage (CT scan or autopsy). Neurological worsening is defined by one or more of the following: 1. Any major change in the level of consciousness. 2. Any substantial change in degree of motor deficit. 3. New clinically significant and persistent deficits. | Within 7 days  Preferably on day 4±2 |
| **Broderick 2013** | An intracranial hemorrhage temporally related to a decline in neurological status as well as new or worsening neurologic symptoms in the judgment of the clinical investigator and which may warrant medical intervention.  A 4 or more point increase in the NIHSS score from baseline to subsequent CT scan at the time of potential worsening could be used as a guide by the clinical investigator for what represents a significant change in neurologic status. | Within 30 hours |
| **Kidwell 2013** | SITS-MOST criteria: PH type 2 hemorrhage visualized on follow-up imaging study and associated with a 4 or more point worsening on the NIHSS score | Within 7 days |
| **Berkhemer 2015** | ECASS II criteria: Evidence of intracranial hemorrhage on imaging studies with neurologic deterioration defined as an increase of 4 or more points in the score on the NIHSS score | At 5 days |
| **Campbell 2015** | SITS-MOST criteria: as parenchymal hematoma type 2 within 36 hours after treatment combined with an increase on the NIHSS of at least 4 points from baseline or the lowest NIHSS value between baseline and 24h | At 24 hours |
| **Goyal**  **2015** | ECASS II criteria: Defined as intracerebral hemorrhage deemed by the site investigator to be the principal cause of neurologic deterioration. | At 24 hours |
| **Saver**  **2015** | Defined as any PH1, PH2, RIH, SAH or IVH associated with a 4 point or more worsening on the NIHSS score within 24 h. | At 27 hours |
| **Jovin**  **2015** | SITS-MOST criteria: Parenchymal hemorrhage type 2 on follow-up imaging and neurologic deterioration of at least 4 points on the NIHSS  ECASS II criteria: Any type of intracerebral hemorrhage on post-treatment imaging with an increase of at least 4 points on the NIHSS. | At 90 days |
